# Signaling pathway dysregulation in breast cancer

**DOI:** 10.18632/oncotarget.28701

**Published:** 2025-03-13

**Authors:** Dinara Ryspayeva, Attila A. Seyhan, William J. MacDonald, Connor Purcell, Tyler J. Roady, Maryam Ghandali, Nataliia Verovkina, Wafik S. El-Deiry, Martin S. Taylor, Stephanie L. Graff

**Affiliations:** ^1^Laboratory of Translational Oncology and Experimental Cancer Therapeutics, Warren Alpert Medical School, Brown University, RI 02903, USA; ^2^Department of Pathology and Laboratory Medicine, Warren Alpert Medical School, Brown University, RI 02903, USA; ^3^Joint Program in Cancer Biology, Lifespan Health System and Brown University, RI 02903, USA; ^4^Legorreta Cancer Center at Brown University, RI 02903, USA; ^5^Pathobiology Graduate Program, Brown University, RI 02903, USA; ^6^Brown Center on the Biology of Aging, Brown University, RI 02903, USA; ^7^Department of Medicine, Hematology/Oncology Division, Lifespan Health System and Brown University, RI 02903, USA

**Keywords:** breast cancer, oncogenic pathways, signal dysregulation in cancer, therapeutic approaches, clinical trials

## Abstract

This article provides a comprehensive analysis of the signaling pathways implicated in breast cancer (BC), the most prevalent malignancy among women and a leading cause of cancer-related mortality globally. Special emphasis is placed on the structural dynamics of protein complexes that are integral to the regulation of these signaling cascades. Dysregulation of cellular signaling is a fundamental aspect of BC pathophysiology, with both upstream and downstream signaling cascade activation contributing to cellular process aberrations that not only drive tumor growth, but also contribute to resistance against current treatments. The review explores alterations within these pathways across different BC subtypes and highlights potential therapeutic strategies targeting these pathways. Additionally, the influence of specific mutations on therapeutic decision-making is examined, underscoring their relevance to particular BC subtypes. The article also discusses both approved therapeutic modalities and ongoing clinical trials targeting disrupted signaling pathways. However, further investigation is necessary to fully elucidate the underlying mechanisms and optimize personalized treatment approaches.

## INTRODUCTION

Breast cancer (BC) is one of the most common cancers in women and a leading cause of cancer-related deaths both in the US and globally [1, 2]. In 2022, over 2 million new BC cases were reported, along with 650,000 deaths, making BC the most prevalent malignant tumor worldwide [3].

BC is a heterogeneous disease comprising several major molecular subtypes [4]. It is well established that BC can be classified based on the expression of estrogen receptor (ER), progesterone receptor (PR), and human epidermal growth factor receptor 2 (HER2) into clinical subtypes including hormone receptor positive, HER2-negative (luminal A or luminal B on molecular testing), HER2-overexpressing (HER2+), or BC negative for ER, PR, and HER2 (ER-/PR-/HER2-), often referred to as triple-negative breast cancer (TNBC), most consistent with the basal subtype on molecular testing. Identifying molecular subtypes is a major step toward the selection of the treatment strategy and prediction of the treatment outcome.

The treatment landscape for BC includes surgery, chemotherapy, radiotherapy, endocrine therapy (ET), targeted therapy (TT), and immunotherapy, requiring collaboration among various subspecialties [5, 6]. Advances in therapeutic approaches have expanded the treatment options for patients with both metastatic and early-stage BC. As of December 2023, the U.S. Food and Drug Administration (FDA) has approved 86 drugs for BC treatment, including chemotherapy agents, TT, and immune checkpoint inhibitors (*NCI. Drugs Approved for Breast Cancer*. Available from: https://www.cancer.gov/about-cancer/treatment/drugs/breast).

The complexity and heterogeneity of tumors underscore the importance of precision medicine in cancer therapy. Expanding the range of targeted molecular alterations can enhance treatment efficacy [7, 8].

Key challenges in treating BC include issues related to both *de novo* and acquired resistance to systemic treatments. This resistance often arises from the dysregulation of signaling pathways within cancer cells, complicating treatment efforts [9, 10]. BC progression involves disruptions in various intra- and intercellular signaling pathways within normal mammary tissues and their surrounding microenvironment. Oncogenic mutations or abnormal expression of signaling components disturb these regulatory networks, leading to uncontrolled tumor cell proliferation, evasion of apoptosis, and tissue invasion [11].

A comprehensive understanding of these dysregulated and dynamic signaling pathways can greatly enhance our knowledge of tumor pathophysiology and guide the development of improved targeted cancer therapies. This review explores the critical roles of various signaling pathways in breast tumor development.

## PI3K/AKT/MTOR PATHWAY

**Figure 1:**
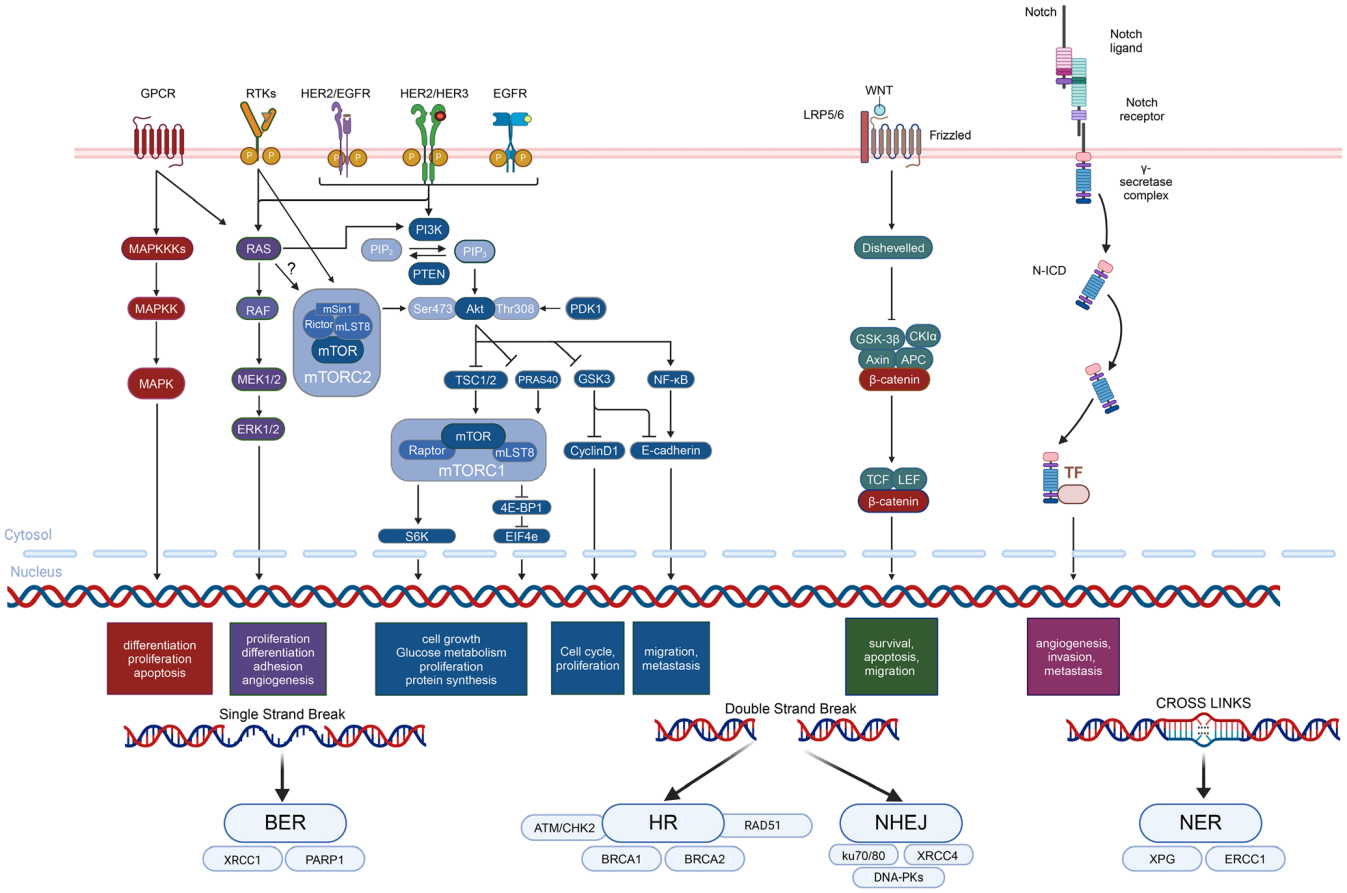
Key signaling pathways and targeted factors in dysregulated BC. This figure illustrates the major signaling pathways and their intricate crosstalk in BC, focusing on the GPCR, RTKs, RAS/RAF/MEK1/2/ERK1/2, MAPK, HER2, PI3K/Akt/mTOR, Wnt/β-catenin, NF-κB, Notch, and DDR pathways. These pathways are critical in regulating essential cellular processes such as proliferation, survival, differentiation, and metastasis. The MAPK pathway, initiated by RTKs like EGFR and HER2, activates downstream effectors such as RAS, RAF, MEK, and ERK1/2, driving cell growth and survival. HER2 amplification further drives oncogenesis by activating the MAPK and PI3K/Akt pathways, with HER3 and HER4 modulating these signals. The PI3K/Akt/mTOR pathway controls cell growth and metabolism through Akt activation and downstream targets like mTORC1/2, p70S6K1, and 4E-BP1. Mutations in PIK3CA and loss of PTEN contribute to its hyperactivation. The Wnt/β-catenin pathway promotes EMT and metastasis, driven by Wnt signaling through LRP5/6 and β-catenin, which interact with APC, CKIα, and TCF/LEF. The NF-κB pathway is a central signaling network regulating inflammation, immune responses, and cell survival. This pathway is activated through Tumor Necrosis Factor Receptors (TNFR), Toll-like Receptors (TLRs), and the IκB kinase (IKK) complex. The Notch pathway, triggered by receptor-ligand interactions between Notch receptors and ligands like DLL, governs cell fate determination and contributes to cancer progression by promoting proliferation and maintaining cancer stem cells. The DDR pathway is essential for maintaining genomic stability by detecting and repairing DNA damage. It responds to various types of damage, including DSBs, SSBs, and cross-links, by activating repair mechanisms such as HR for DSBs and NER to other kinds of damage. In BC, dysregulation of the DDR pathway, often due to mutations in genes like BRCA1 and BRCA2, impairs DNA repair, leading to genomic instability and an increased risk of cancer.

The PI3K/Akt/mTOR signaling pathway (Figure 1) is pivotal in regulating cell growth, proliferation, metabolism, and survival [12–14]. Up to 25–40% of BC cases exhibit variations that hyperactivate the PI3K/Akt/mTOR pathway, underscoring its critical role in oncogenesis [15–17].

### PIK3CA gene mutation

PI3K produces the phospholipid phosphatidyl-3,4,5-triphosphate (PIP_3_) in the inner leaflet of the plasma membrane by phosphorylating Phosphatidylinositol 4,5-bisphosphate (PIP_2_) and is the first step in the most frequently altered pathway in BC. Mutations and amplifications commonly occur in the genes encoding the PI3K catalytic subunits p110α (PIK3CA) and p110β (PIK3CB) [12, 16, 18, 19]. These genetic alterations lead to constitutive activation of the PI3K pathway, which drives oncogenic processes such as cell growth, survival, and proliferation. This pathway plays a central role in ER-positive BCs.

PIK3CA mutations were found in 32% of early BC patients and were associated with favorable clinicopathologic characteristics, such as older age, ER positivity, lower grade, and smaller tumor size [20]. The prevalence of PIK3CA mutations is 18% in TNBC, 22% in HER2-positive, and 37% in luminal subtypes [21].

It is important to note that these findings are different from reports linking PI3K pathway activation to resistance to ET [22, 23]. Although PIK3CA mutations can occur throughout the gene, up to 80% of PIK3CA mutations occur in hotspots within the helical (E542K and E545K) and kinase (H1047R) domains of p110α. These mutations significantly increase PI3K activity, leading to the induction of cellular transformation *in vitro* and tumorigenicity *in vivo* when overexpressed in human mammary epithelial cells. Moreover, transgenic mice expressing these mutant forms of p110α develop mammary tumors, highlighting their oncogenic potential. The mutations more frequent in tumors that relapse during ongoing ET (48%). The hyperactivation of the PI3K pathway, often driven by these mutations, is associated with resistance to ET in BC [22, 23].

### Loss of PTEN

PTEN (Phosphatase and Tensin Homolog) is a tumor suppressor that negatively regulates the PI3K/Akt/mTOR pathway by dephosphorylating PIP_3_ back to PIP_2_, thus inhibiting Akt activation [24, 25].

Loss of PTEN function, whether due to mutations, deletions, or epigenetic silencing, removes this regulatory brake, resulting in sustained pathway activation and cancer progression [24].

Transcription of the PTEN gene is regulated at multiple levels [24–28]. Epigenetic and transcriptional positive regulation involves factors such as early growth response protein-1 (EGFR-1), peroxisome proliferator-activated receptor-γ (PPAR-γ), tumor protein 53 (p53), human sprout homolog 2 (SPRY2) and activating transcription factor 2 (ATF2). Transcriptional regulation is also controlled by negative regulators, including c-Jun, nuclear factor kappa B (NF-κB), transforming growth factor beta (TGF-β), and the polycomb group protein BMI1 [24, 29, 30]. Additionally, SNAIL and SLUG repress PTEN expression at the transcriptional level. Epigenetic mechanisms, including promoter methylation and histone modifications, can also lead to the silencing of PTEN expression [24].

At the post-transcriptional level, various miRNAs have been identified that downregulate PTEN expression [24, 26, 28, 31]. Additionally, post-translational modifications of PTEN by SUMOylation [32], phosphorylation of the C-terminal tail [33, 34], acetylation [35, 36], ubiquitination [37, 38] and other novel modifications were described in review [39].

The PTEN promoter has been described as a potential target in BC [40–45]. Research indicates that loss of PTEN may predict more aggressive disease and poorer outcomes in patients with BC and is more commonly observed in TNBC [46–48]. The loss of PTEN contributes to disease progression and resistance to TT by driving activation of the PI3K pathway, as well as functional “cross-activation” of the MAPK pathway [49].

### Akt amplification and mutation

The serine/threonine protein kinase Akt also referred to as protein kinase B (PKB) protein kinase B (PKB), is activated by the PI3K pathway by binding to PI3K-produced PIP_3_ through its N-terminal Pleckstrin homology (PH) domain at the plasma membrane [14, 50]. Akt (which we use to refer to Akt1) is subsequently phosphorylated by mTORC2 on its hydrophobic motif Ser473, relieving autoinhibition and permitting subsequent phosphorylation at Thr308 in the activation loop by PDK1 and resultant full activation [51–54]. The Akt kinase family consists of three homologous and highly similar isoforms: Akt1 (PKBα), Akt2 (PKBβ) and Akt3 (PKBγ). Akt1 plays a critical role in multiple cellular processes, including growth, survival, and metabolism, while Akt2 is central to maintaining glucose homeostasis. Akt3 is primarily involved in neuronal development [55].

Alterations in the *AKT* gene, such as amplification or mutation, can enhance Akt kinase activity, which is linked to worse survival outcomes, especially in ER-negative BC [56, 57]. Mutations in *AKT1*, *AKT*2, or *AKT*3 are found in roughly 3–5% of cancers, with the most common functionally activating *AKT* mutations being E17K, L52R, and Q79K [58–62]. *AKT* mutations are frequently observed in hormone-driven cancers, particularly in ER-positive BC subtypes [63].

In BC the three Akt isoforms play distinct roles in regulating migration and other cellular functions [50, 64–67]. Akt1 is primarily responsible for the proliferative potential of cells by upregulating Cyclin D1 and S6 and is more highly expressed in the primary BC tumor sample from the breast. *AKT1* E17K is the most frequently identified oncogenic mutation in Akt1 [61]. In contrast, Akt2 expression is elevated in lung and liver metastatic samples, where it is associated with increased invasiveness, stem cell-like characteristics, and resistance to therapies [65, 66]. Akt3 is mainly amplified and highly expressed in TNBC tumors and cell lines, where it plays a crucial role in regulating tumor growth and progression [64, 68, 69].

### mTOR activation

The mammalian target of rapamycin (mTOR) is a 289 kDa serine/threonine kinase that plays a key role in regulating cell growth and metabolism. Its activation can occur due to mutations in upstream components or alterations in nutrient and energy-sensing mechanisms [70, 71]. The mTOR signaling cascade comprises two distinct multi-subunit complexes: mTORC1 and mTORC2, each formed by the mTOR kinase associating with different adapter proteins [72, 73].

The large megadalton mTORC1 and mTORC2 complexes were identified as assemblies of multiple proteins, as shown in Table 1. They differ based on the binding of (Raptor) or (Rictor and mSin1) to (mTOR+mLST8), which leads to the formation of mTORC1 and mTORC2, respectively [72]. The two complexes have distinct and sometimes opposing functions and feedback loops; a simplified summary is that mTORC1 on the lysosome surface synthesizes signals from nutrients and stress to promote anabolism vs catabolism, and mTORC2 at the plasma membrane synthesizes signals from outside the cell including various growth factors such as insulin to control growth and metabolism [74].

**Table 1:** mTORC1 and mTORC2 complexes

mTORC1	mTORC2
mTOR protein	mTOR protein
Raptor	Rictor
GβL/mLST8	GβL/mLST8
DEPTOR	DEPTOR
PRAS40	Protor/PRR5
	mSIN1

Aberrant mTOR signaling is a hallmark of many cancers and is associated with increased tumor progression [75]. The activation of the mTOR pathway (Figure 1) depends on activating mutations in mTOR, mTORC1/2, or upstream mutations, as well as the loss of function of negative regulators in the mTOR signaling cascade [73, 76].

Mutations in core components of the mTOR complexes (mTORC1 and mTORC2) are rarer than the more common upstream mutations in pathways such as Akt and TSC1/2 (tuberous sclerosis complex proteins). These mutations often result in the overactivation of mTORC1, which is crucial for cancer progression and resistance to therapy [77].

mTORC1 plays a central role in maintaining the balance between anabolic and catabolic processes, especially in response to environmental stress. TSC1 and TSC2 are crucial negative regulators of mTORC1. They inhibit mTORC1 activity by transforming the small GTPase Rheb (Ras homolog enriched in the brain) into its inactive GDP-bound state. This mechanism prevents uncontrolled cell growth and proliferation [70, 78]. mTORC1 is also controlled and regulated at the lysosome surface by the two small heterodimeric GTPases RagA/B and RagC/D, forming a bipartite switch modulated by numerous proteins including the tumor suppressor FLCN; together these three GTPases tightly control mTORC1 activity [74, 79–83].

TSC1 and TSC2 are tumor suppressors, and their loss or mutation causes widespread but benign tumors. In TSC mutant cells, mTORC1 is constitutively active, therefore stimulating translation and promoting cell growth [84]. Low expression of TSC 1/2 is associated with more aggressive BC and worse outcomes [85]. TSC2 normally inhibits mTORC1 by promoting the conversion of Rheb-GTP to its inactive form, Rheb-GDP. When TSC2 is lost or its function is impaired, this conversion is hindered, resulting in elevated Rheb-GTP levels and subsequent activation of mTORC1 [14, 71, 86].

There are a variety of upstream pathways that control mTORC1 activation, including growth factor signaling, amino acid levels, cellular energy levels, and stress (in review [70, 71, 74]. Akt modulates mTORC1 activity by phosphorylating PRAS40, a key inhibitor of mTORC1. This phosphorylation removes PRAS40’s inhibition, thereby enhancing mTORC1 activation [87]. The Ras-Erk MAPK pathway can also activate mTORC1 downstream. When Erk is activated, it directly phosphorylates and inactivates TSC2.

mTORC2 is less well understood than mTORC1 but acts as a regulated effector of IGF and PI3K signaling [71, 74]. It also appears to modulate a portion of signaling downstream of oncogenic Ras [88, 89]. Rictor, a core component of mTORC2, is sometimes highly amplified in patients with lung cancer and BC. In addition to activation of Akt, mTORC2 activates numerous members of the AGC kinase family including PKC, PKN, and SGK controlling metabolism, cell division, and migration [72, 90]. Intriguingly, SGK may substitute for Akt activity as a resistance mechanism in Akt inhibition [91, 92]. Development of selective mTORC2 inhibitors could prevent such a mechanism, but selective inhibitors of mTORC2 vs mTORC1 are not available and have been challenging to make because both complexes include mTOR as the key active component in essentially identical conformations.

### Therapeutic approaches

Given the critical role of the PI3K/Akt/mTOR pathway in cancer, numerous therapeutic strategies have been devised to target various components of this pathway. Targeting the PI3K/Akt/mTOR signaling pathways has led to the development of drugs that address mechanisms of endocrine resistance [93].

### PI3K inhibitors

Numerous PI3K inhibitors in clinical development inhibit all catalytic subunit isoforms, such as p110α, p110β, and p110δ. However, some inhibitors are designed to target only specific isoforms [94]. Despite challenges such as poor drug tolerance and resistance, several PI3K inhibitors have now received regulatory approval (Table 2) [95].

**Table 2:** Approved drugs for BC treatment

Agents		Trial	FDA	Indication and disease setting	References
**PI3K Inhibitors**					
Alpelisib	p110α-selective inhibitor	Phase III, SOLAR-1 (NCT02437318)	Approved 2019	PIK3CA-mutated ER/PR-positive, HER2-negative mBC	(André et al., 2019) [96]
Inavolisib	p110α-selective degrader	Phase III, INAVO120 (NCT04191499)	Approved 2024	PIK3CA-mutated HR-positive, HER2-negative, locally advanced or mBC	(Turner et al., 2024) [98]
**Akt Inhibitors**					
Capivasertib	Akt-inhibitor	Phase III, CAItello-291 (NCT04305496)	Approved 2023	ER/PR-positive, HER2-negative mBC with PIK3CA/Akt1/PTEN-alterations	(Turner et al., 2023) [102]
**mTOR Inhibitors**					
Everolimus	mTOR inhibitor	Phase III, BOLERO-2 (NCT00863655)	Approved 2012	ER/PR-positive, HER2- negative mBC	(Baselga et al., 2012) [105]
**Anti-HER2 therapeutic agents**					
Trastuzumab	Anti-HER2 mAb	Phase III	Approved 1998	HER2+ mBC	(Cobleigh et al., 1999) [320]
		Phase III, HERA (NCT00045032)	Approved 2006	HER2+ eBC	(Piccart-Gebhart et al., 2005) [321]
Pertuzumab	Anti-HER2 mAb	Phase III, APHINITY (NCT01358877)	Approved 2017	HER2+ eBC in combination with trastuzumab and chemotherapeutic agents	(von Minckwitz et al., 2017) [156]
		Phase III, CLEOPATRA (NCT00567190)	Approved 2012	HER2+ mBC in combination with trastuzumab and docetaxel	(Swain et al., 2013) [322]
Lapatinib	HER1/HER2 TKI inhibitor	Phase III, (NCT00078572)	Approved 2007	HER2+ mBC in combination with capecitabine	(Geyer et al., 2006) [323]
Neratinib	HER1/HER2/HER4 TKI inhibitor	Phase III, NALA (NCT01808573)	Approved 2020	HER2+ mBC after receiving 2 or more anti-HER2 based treatment regimens	(Saura et al., 2020) [158]
		Phase III, ExteNET (NCT00878709)	Approved 2017	HER2-overexpressed/amplified eBC, to follow adjuvant trastuzumab-based therapy	(Martin et al., 2017) [324]
Tucatinib	HER2/HER3 TKI inhibitor	Phase II, HER2CLIMB (NCT02614794)	Approved 2020	HER2+ mBC in combination with trastuzumab and capecitabine	(Murthy et al., 2020) [157]
Ado-trastuzumab emtansine (T-DM1)	Anti-HER2 monoclonal anti-microtubule agent conjugate	Phase III, EMILIA (NCT00829166)	Approved 2013	HER2+ mBC after trastuzumab and a taxane	(Verma et al., 2012) [152]
		Phase III, KATHERINE (NCT01772472)	Approved 2019	HER2+ eBC with residual invasive disease after neoadjuvant taxane and trastuzumab	(von Minckwitz et al., 2019) [325]
Fam-trastuzumab deruxtecan-nxki (T-DXd)	Anti-HER2 monoclonal topoisomerase inhibitor conjugate	Phase III, DESTINY-Breast03 (NCT03529110)	Approved 2022	HER2+ mBC after receiving an anti-HER2 based treatment regimen	(Cortés et al., 2022) [163]
		Phase III, DESTINY-Breast04 (NCT03734029)	Approved 2022	Previously treated HER2-Low mBC	(Modi et al., 2022) [326]
Margetuximab-cmkb	Fc-engineered anti-HER2 mAb	Phase III, SOPHIA (NCT02492711)	Approved 2020	HER2+ mBC after receiving two or more anti-HER2 based treatment regimens	(Rugo et al., 2023) [327]
**PARP Inhibitors**					
Olaparib	PARP1/PARP2 inhibitor	Phase III, OlympiA (NCT02032823)	Approved 2022	BRCA1 or BRCA2 germline mutations high-risk HER2-negative eBC	(Tutt et al., 2021) [297]
		Phase III, OlympiAD (NCT02000622)	Approved 2018	BRCA1 or BRCA2 germline mutations HER2-negative mBC after receiving no more than two previous chemotherapy regimens for metastatic disease	(Robson et al., 2017) [298]
Talazoparib	PARP1/PARP2 inhibitor	Phase III, EMBRACA (NCT01945775)	Approved 2018	germline mutations in BRCA1 and BRCA2 HER2-negative mBC	(Litton et al., 2018) [328]

Abbreviations: eBC: early-stage breast cancer; mBC: metastatic breast cancer.

Alpelisib is a p110α-selective inhibitor, approved for the treatment of PIK3CA-mutated ER/PR-positive, HER2-negative BC in 2019 [96].

Taselisib, a p110β-sparing inhibitor, demonstrated statistical improvement in PFS in the phase III study, but with serious side effects and was not approved by the FDA [16, 18].

Inavolisib, a p110α-selective degrader, was approved by the FDA on October 10, 2024, based on the results of the INAVO120 trial [97, 98]. It continues to be developed in early-stage BC treatment (Table 3, NCT05306041).

**Table 3:** Current clinical trials targeting BC: progress and emerging therapies

Drug name	Disease indications tested in trials	Interventions	Development phase	Primary outcome measures	Clinicaltrials.gov identifier
***PI3K inhibitors***					
PI3Kα-selective inhibitor alpelisib	ER+/HER2- mBC	Alpelisib Palazestrant Ribociclib Everolimus	Phase Ib	DLTs MTD	NCT05508906
PI3Kα selective inhibitor inavolisib	ER+/HER2+ PIK3CA mutant eBC	Inavolisib PHESGO Endocrine therapy	Phase II	pCR rate (ypT0/is ypN0)	NCT05306041
PI3Kα H1047R mutation selective inhibitor OKI-219	Advanced solid tumors with PI3Kα H1047R mutation, including mBC	OKI-219 Fulvestrant Trastuzumab	Phase Ia/Ib	MTD	NCT06239467
Covalent inhibitor of PI3Kα mutation TOS-358	Advanced solid tumors, including BC	TOS-358	Phase I	DLTs RP2D	NCT05683418
Allosteric PI3Kα inhibitor STX-478	Advanced solid tumors with PI3Kα mutations	STX-478 Fulvestrant Ribociclib Palbociclib	Phase I/II	DLT ORR	NCT05768139
PI3K δ-sparing inhibitor MEN1611	ER+/HER2- mBC with PIK3CA/PTEN-alterations	MEN1611 Eribulin	Phase II	CBR ORR	NCT05810870
Dual PI3K δ/γ inhibitor tenalisib	mTNBC	Tenalisib (RP6530)	Phase II	CBR ORR PFS	NCT06189209
Pan-PI3K/mTOR inhibitor Gedatolisib	ER+/HER2- mBC	Gedatolisib Palbociclib Fulvestrant Alpelisib	Phase III	PFS	NCT05501886
Pan-PI3K and mTOR inhibitor GDC-0084	HER2+ mBC with brain metastases	GDC-0084 Trastuzumab	Phase II	CNS-ORR	NCT03765983
PI3Kα:RAS breaker BBO-10203	Advanced solid tumors (BREAKER-101)	BBO-10203 Trastuzumab	Phase I	MTD AEs RP2D	NCT06625775
***Akt inhibitors***					
Ipatasertib	HER2+ mBC with PI3KCA-mutations	Ipatasertib Trastuzumab Pertuzumab	Phase Ib	RP2D	NCT04253561
Ipatasertib	eBC (TNBC) with and without PI3CA/AKT1/PTEN genetic alterations	Ipatasertib Atezolizumab Chemotherapy	Phase I	pCR	NCT05498896
Ipatasertib	mBC (activating Akt mutations) The ComboMATCH treatment trials (cohort EAY191-S3)	Ipatasertib Paclitaxel	Phase II	Accrual and assignment of patients Enrollment rates to trial	NCT05564377
***mTOR inhibitors***					
Dual mTORC1/2 inhibitor vistusertib (AZD2014)	mTNBC	Vistusertib Olaparib	Phase Ib/II	MTD	NCT02208375
Vistusertib (AZD2014)	ER+ mBC	Vistusertib Fulvestrant	Phase I	AEs Pharmacokinetics	NCT01597388
***MEK inhibitors***					
Cobimetinib	Inflammatory mBC	Atezolizumab + Cobimetinib + Eribulin.	Phase II	ORR	NCT03202316
Selumetinib	mTNBC (arm II)	Olaparib Selumetinib	Phase II	ORR	NCT03801369
Binimetinib	EAY191-N2 (NF1 mutations) (ComboMATCH Trial)	Binimetinib Fulvestrant	Phase II	Accrual and assignment of patients Enrollment rates to trial	NCT05564377
***Anti-HER2 TKIs***					
Early-stage HER2+ BC
Pyrotinib	HER2+ microinvasive eBC (stage I)	Pyrotinib plus capecitabine (adjuvant therapy)	Phase II	iDFS	NCT05861271
Pyrotinib	HER2+ eBC	Pyrotinib (extended adjuvant therapy)	Phase II	iDFS	NCT05880927
Pyrotinib	HER2+ high risk eBC	Pyrotinib after adjuvant trastuzumab combined with pertuzumab or T-DM1	Phase II	iDFS	NCT05834764
Pyrotinib	Residual invasive HER2+ eBC	Pyrotinib after neoadjuvant chemotherapy plus anti-HER2 therapy	Phase II	iDFS	NCT04254263
Pyrotinib	HER2+ eBC (neoadjuvant therapy)	Pyrotinib combined with trastuzumab and chemotherapy	Phase II	pCR	NCT04481932
Metastatic setting					
Pyrotinib	HER2+ mBC	Pyrotinib combined with trastuzumab and chemotherapy in the first-line setting	Phase II	PFS	NCT05429294
Pyrotinib	HER2+ mBC with active brain metastases after ADCs	Pyrotinib plus capecitabine	Phase II	CNS-ORR	NCT06475443
Pan-HER receptor TKI Neratinib	HER2- mBC with brain metastasis and abnormally active HER2 signaling	Neratinib and Capecitabine	Phase II	OS CNS-PFS	NCT04965064
Neratinib	Stage I-III HER2+ eBC with detected molecular residual disease	Neratinib and T-DM1 (adjuvant therapy)	Phase II	Clearance of ctDNA with the addition of neratinib to T-DM1	NCT05388149
Neratinib	HER2+ mBC with brain metastases (Cohort 1, 4)	Neratinib T-DM1	Phase II	ORR CNS-ORR	NCT01494662
Neratinib	HER2+ mBC	Neratinib Capecitabine	Phase Ib/II	MTD	NCT03377387
Ibrutinib	HER2-amplified mBC in the setting of T-DM1-pretreated disease	Trastuzumab plus Ibrutinib	Phase I/II	MTD CBR	NCT03379428
***HER2-Targeting ADC***					
BB-1701	HER2+ or HER2-low mBC	BB-1701 (ADC)	Phase II	AEs ORR	NCT06188559
SHR-A1811	HER2+ eBC Neoadjuvant Treatment	SHR-A1811 Pyrotinib	Phase II	pCR	NCT05635487
ARX788	HER2+ eBC after treatment with trastuzumab and pertuzumab.	Pyrotinib ARX788	Phase II	RCB	NCT04983121
	HER2+ mBC after treatment T-DXd	ARX788	Phase II	ORR	NCT04829604
	HER2+ mBC after T-DXd therapy	ARX788	Phase II	ORR	NCT06578286
IKS014	HER2+ advanced solid tumors, including BC	IKS014	Phase I	RP2D ORR	NCT05872295
FS-1502	HER2+ mBC, HER2 expressed advanced solid tumors	FS-1502	Phase 1	DLT MTD RP2D ORR	NCT03944499
	HER2+ mBC	FS-1502 versus T-DM1	Phase III	PFS	NCT05755048
GQ1001	HER2+ mBC	GQ1001	Phase I	DLT MTD RP2D	NCT04450732
	HER2+ mBC after previous anti-HER2 treatment	GQ1001+ pyrotinib	Phase Ib/II	DLT MTD AEs ORR	NCT05575804
Degrader-antibody conjugate ORM-5029	HER2+ mBC and advanced solid tumors	ORM-5029	Phase I	MTD AEs ORR DOR	NCT05511844
***KRAS inhibitor***					
KRAS G12C Inhibitor Adagrasib (MRTX849)	KRAS G12C Mutated Advanced Solid Tumors, including BC	Adagrasib Olaparib	Phase Ib	AEs	NCT06130254
***Bispecific antibody (BsAb)***					
Anti-HER2/SIRPα BsAb IMM2902	HER2+ mBC	IMM2902	Phase I	DLT MTD AEs Toxicity	NCT05076591
Anti-SIRPα BsAb DS-1103a	HER2+ advanced solid tumors	DS-1103a	Phase I	DLT AEs ORR	NCT05765851
HER2-targeting BsAb Zanidatamab	HER2+ mBC	Zanidatamab	Phase III	PFS	NCT06435429
***Vaccines***					
Multi-epitope HER2 peptide vaccine TPIV100	Stage II-III HER2+ eBC with residual disease post-neoadjuvant chemotherapy	TPIV100	Phase II	iDFS	NCT04197687
HER2/neu peptide vaccine GLSI-100	HLA-A*02 positive and HER2+ high risk eBC	GLSI-100	Phase III	Invasive Breast Cancer-free Survival	NCT05232916
WOKVAC vaccine	HER2+ eBC	pUMVC3-IGFBP2-HER2-IGF1R plasmid DNA Vaccine + anti-HER2-mAbs	Phase II	TILs	NCT04329065
HER-2 Directed dendritic Cell (DC1)	HER2+eBC	HER-2 pulsed DC1 Trastuzumab Pertuzumab	Phase II	pCR rate Immunogenicity	NCT05325632
Dendritic cell vaccine	HER2- mBC	CircFam53B-219aa DC vaccine	Phase I	DLT AEs	NCT06530082
Dendritic cell vaccines against HER2/HER3	HER2+ BC or TNBC with brain metastasis	Anti-HER2/HER3 dendritic cell vaccine Pembrolizumab	Phase II	CNS - ORR	NCT04348747
***Target DDR pathway***					
Selective PARP1 inhibitor saruparib (AZD5305)	Advanced solid cancers	AZD5305	Phase I/IIa	AEs DLT	NCT04644068
Selective PARP1 inhibitor saruparib (AZD5305)	ER+/HER2- mBC with BRCA1, BRCA2, or PALB2 mutations (arm 1)	Saruparib Camizestrant	Phase III	PFS	NCT06380751
Selective PARP1 inhibitor HRS-1167	gBRCA1/2 HER2- eBC (neoadjuvant therapy)	HRS-1167	Phase II	DLT AEs pCR rate	NCT06516289
DNA polymerase (pol) theta inhibitor ART6043	HER2-ve mBC, g/sBRCA mutations	ART6043	Phase I/IIa	DLT PFS	NCT05898399
ATR Inhibitor M1774	ER+/HER2- mBC after CDK4/6 inhibitors	M1774 Fulvestrant	Phase I/II	DLT	NCT05986071
HDAC Tucidinostat	ER+/HER2- mBC	Tucidinostat	Phase 2	ORR	NCT05633914

Abbreviations: ADC: antibody-drug conjugate; AEs: adverse events; CBR: clinical benefit rate; CNS: central nervous system; DLTs: dose limiting toxicities; DLTs: dose limiting toxicities; DOR: duration of response; MTD: maximum tolerated dose; MTD: maximum tolerated dose; ORR: overall response rate; OS: overall survival; pCR: pathologic complete response; PFS: progression free survival; RCB: residual tumor burden classification in grades; RP2D: recommended phase II dose; TILs: tumor-infiltrating lymphocytes.

The pan PI3K inhibitor, pictilisib did not meet its primary endpoint in the PEGGY trial (ER+/HER2 negative BC, NCT01740336) [99].

Other PI3K inhibitors, such as TOS-358, MEN1611, OKI-219, STX-478, BBO-10203 and tenalisib are currently being investigated for BC in clinical trials (Table 3). Additionally, PI3K inhibitors, such as idelalisib, copanlisib, duvelisib, and umbrasilib are used in hematological malignancies.

Different PIK3CA mutations might have distinct prognostic implications. The importance of multiple PIK3CA mutations is evaluated in trials (NCT04632992; NCT04589845, NCT05564377).

PI3K/Akt signaling pathway is overactivated in many human cancers, leading to excessive DNA damage response activation [100]. PTEN loss contributes to this issue by causing resistance to PI3Kα inhibitors. Additionally, PTEN loss is a mechanism of acquired resistance to CDK4/6 inhibitors. Clinically, PTEN loss is relevant because it also reduces the effectiveness of PI3Kα inhibitors, which are currently used after CDK4/6 inhibitors [101].

### Akt inhibitors

Clinical trials are currently exploring the use of Akt inhibitors, either as single agents or combined with other treatments, to address the oncogenic effects caused by Akt1 activation (Table 3, NCT03959891, NCT04253561 NCT05564377).

The findings suggest that different Akt mutants exhibit varying sensitivities to Akt inhibitors [61]. Drugs like capivasertib [102] and ipatasertib [103] block Akt kinase activity, targeting its pro-survival effects to improve outcomes in cancers driven by Akt mutation or hyperactivity.

Ipatasertib, an ATP-competitive selective Akt inhibitor, is currently being evaluated in clinical trials for its efficacy in BC (Table 3). While it did not demonstrate improved outcomes in PIK3CA/Akt1/PTEN-altered advanced TNBC [104], it has shown promising results in patients with HER2-positive mBC harboring PIK3CA mutations, according to preliminary findings from the phase 1b IPATHER trial (NCT04253561).

Capivasertib is a pan-Akt small-molecule inhibitor of all three Akt isoforms approved for the treatment of ER/PR-positive BC that is also has either PIK3CA-mutated or Akt-altered or demonstrated PTEN-loss (Table 2) [102].

### mTOR inhibitors

mTOR inhibitors, such as everolimus [105] and temsirolimus, are used in the treatment of various cancers, including renal cell carcinoma and BC. These drugs acutely inhibit mTORC1, leading to reduced protein synthesis and cell proliferation. However chronic therapy also inhibits mTORC2, and side effects include a diabetes-like state due to loss of insulin signaling [74].

AZD2014, also known as vistusertib, is a potent small-molecule ATP-competitive inhibitor of mTOR that selectively targets both mTORC1 and mTORC2 complexes. This dual inhibition provides a broader scope of action by addressing not only tumor growth but also resistance mechanisms that may arise due to mTORC2 activity. Preclinical studies have demonstrated that AZD2014 induces significant tumor regression, particularly in ER+ mBC [106]. It is currently being evaluated in clinical trials to further assess its therapeutic potential and efficacy in combination with other targeted therapies, with the goal of improving outcomes for patients with advanced BC (Table 3).

Dual inhibitors that target both PI3K and mTOR provide a more comprehensive blockade of the pathway. For example, gedatolisib aims to overcome resistance mechanisms that can develop when targeting only one component of the pathway (Table 3, NCT05501886). Gedatolisib has shown higher efficacy in BC patient-derived xenograft models [107].

In conclusion, the PI3K/Akt/mTOR pathway remains a cornerstone of cancer biology and a critical target for therapeutic intervention. Continued research and clinical trials are essential to fully harness the potential of targeting this pathway, addressing resistance mechanisms, and improving patient outcomes.

## RAS/RAF/MEK/ERK PATHWAY

The RAS/RAF/MEK/ERK pathway, a key signaling axis often altered in cancer, is typically considered to be an infrequently mutated pathway in BC [108].

However, high-fidelity molecular techniques have uncovered the critical relevance of non-genetic RAS/RAF/MEK/ERK pathway activation in BC, enabling the effective use of advanced TT. This pathway is activated by oncogenic mutations, as well as epigenetic and transcriptional regulation not captured by genetic sequencing alone. The variability in RAS/RAF/MEK/ERK pathway alterations presents a major challenge in treating BC.

The RAS/RAF/MEK/ERK pathway consists of a GTPase and three layers of protein kinases that sequentially phosphorylate each other, transmitting extracellular signals to the cell nucleus where they influence cell division, differentiation, and survival [109]. The pathway is initiated when a cell surface receptor, such as a receptor tyrosine kinase (RTK), G-protein coupled receptor (GPCR), hormone receptor, or interleukin receptor, binds to its corresponding growth factor. Most commonly, this pathway is activated by ligand binding to an RTK, which leads to receptor dimerization and autophosphorylation of its intracellular domain. This activation recruits guanine nucleotide exchange factors (GEFs) that activate Ras, a member of the GTPase family. Ras proteins, encoded by the *HRAS, NRAS,* and *KRAS* genes, act as molecular switches; they are tethered to the plasma membrane and activated when GEFs exchange GDP for GTP, moving Ras into an active state. However, because Ras has low enzymatic activity, GTPase-activating proteins (GAPs) accelerate this process. Once activated by GTP, Ras binds to the cytoplasmic RAF kinase, most commonly BRAF, which then dimerizes and activates MEK through phosphorylation. Phosphorylated MEK, in turn, phosphorylates ERK (Figure 1). Activated ERK then triggers various transcription factors, including *ETS1/2*, *ELK1*, and *JUN,* which regulate cell development, migration, and growth [110–112]. This pathway is notable for its significant signal amplification, where one upstream protein can activate multiple downstream effectors. Non-canonical Ras signaling has recently been identified and a portion of oncogenic Ras signaling is conveyed by mTORC2; intriguingly genetic ablation of mTORC2 in a mouse model system of Ras activated melanoma resulted in markedly reduced tumor growth [88].

### RAS/RAF/MEK/ERK pathway aberrations and prognostic impact

This signal amplification significantly worsens the oncogenic effect of this pathway when dysregulated in cancer. In BC, the core RAS/RAF/MEK/ERK genes are rarely mutated, with *KRAS, HRAS, NRAF,* and *BRAF* gene mutation rates of less than 1% across all subtypes [113–116]. Additionally, *NF1,* which hydrolyzes GTP on Ras, thereby deactivating it, shows mutation rates between 3.0% and 3.8% [113, 117]. TNBC stands out as *KRAS* and *BRAF* mutations are observed at rates of 30% and 32%, respectively. In TNBC, these mutations are perhaps incentivized due to the lack of pro-growth signaling from hormone receptors and RTK HER2 [117]. An analysis of 2859 patient samples demonstrated that gene alterations, which include mutations, copy number alterations, and structural variants, were limited to below approximately 2% of patients for the genes *NF1, KRAS, HRAS, BRAF, MAPK1, JUN,* and *RAF1* across BC subtypes. Notable exceptions were *NF1, KRAS*, and *BRAF* alterations in basal-type cancer, as well as increased *NF1* and *KRAS* in HER2-positive disease [118]. In the same study, genetic alterations in the RAS/RAF/MEK/ERK genes were linked to significantly reduced overall patient survival when all BC subtypes were combined.

The RAS/RAF/MEK/ERK is also non-mutationally activated in BC due to overexpression of RTKs, especially in the HER2-positive phenotype [119]. RASAL2, a Ras GTPase-activating protein, shows promoter hypermethylation in 50% of luminal B tumors, reducing overall survival [120].

### HER2+ pathway

HER2 phenotype of BC, also known as ERBB2 or HER2-neu, represented overexpression in tumor cells. This phenotype makes up 20–25% of all BC cases [121, 122], and before HER2 targeting therapies was one of the subtypes with the worst clinical outcomes [123, 124]. Fortunately, modern therapeutics have improved outcomes for HER2+ BC, but there is still more to do especially in the case of advanced metastatic disease [125, 126].

HER2 is a transmembrane protein with tyrosine kinase activity that falls into the epidermal growth factor (EGF) family of receptors; known for their function for stimulating cell growth and differentiation [121, 127–129]. However, unlike other EGF receptors, HER2 cannot function autonomously as it does not bind any growth factors itself, so it must act solely as a coreceptor through heterodimerization with the other 3 receptors found in the ERBB family or through homodimerization with another HER2 molecule [123, 130, 131]. In HER2+ BC dimerization most commonly occurs between the HER2 and HER3 receptors (Figure 1); HER3 can functionally bind ligands but is a catalytically dysfunctional tyrosine kinase so it acts primarily as an allosteric activator of the other family members [132–134]. Both HER2 and HER3 play a synergistic role in HER2+ BC progression and are both being used as targets for therapy [135, 136].

### HER2+ pathway dysregulation in BC

HER2 amplification alone is enough to result in spontaneous receptor dimerization and subsequent phosphorylation without ligand binding [137]. This leads to the constitutive activation of EGFR signaling pathways and their subsequent promotion of tumor progression [138, 139]. The most studied HER2 downstream signaling pathways are the RAS/Raf/Mitogen-activated protein kinase (MAPK) and the PI3K/Akt cascades (Figure 1).

The HER2+ subtype is highly dependent on the activation of the PI3K/Akt pathway for growth and tumor progression [140]. In 31% of all HER2+ tumors PIK3CA is mutated with 69% of those mutations being one of the following: H1047R (35%), E545K (17%), E542K (10%), and H1047L (5%) N345K (2%) and result in the aberrant activation of the PI3K pathway [141]. Mutations in PIK3CA not only drive oncogenesis but also often confer resistance to first-line trastuzumab treatment in BC [142, 143]. In HER2+ BC, PTEN mutations are less common than PIK3CA mutations, occurring in fewer than 10% of treatment-naïve primary tumors [117]. However, trastuzumab itself activates PTEN through the inhibition of Src kinase activity by blocking its association with ERBB2. Due to this pressure, 40% of HER2 overexpressing BC eventually develop PTEN deficiency resulting in primary or acquired resistance to trastuzumab [144, 145]. PI3K inhibitors have been shown to resensitize PI3K and PTEN-altered HER2+ trastuzumab-resistant cells *in vitro* and *in vivo* [146, 147].

As previously described, the RAS/RAF/MEK/ERK pathway remains largely intact in BC [113–116]. This is noticeably true in HER2+ BC due to its overreliance on the PI3K/Akt pathway [140]. Despite this strong reliance, RAS/RAF/MEK/ERK plays an important role in drug resistance to HER2 targeting therapies. This was shown to occur, both *in vitro* and *in vivo*, through a switch to MEK/ERK from PI3K/Akt as the primary driver pathway for tumor progression post-treatment [148]. This switch elicits sensitivity to MEK and ERK inhibitors due to the now strong dependence on the activation of this pathway [148]. Resistance to trastuzumab has also been shown *in vitro* to be acquired through the drug-mediated upregulation and autocrine production of CCL5 and eventual constitutive activation of ERK and NFkB. MEK inhibition and CCR5 antagonism partially reverse this trastuzumab resistance and may offer good therapeutic targets for resensitizing tumors in the case of cancer progression after first-line treatments [149]. Despite playing a smaller role in overall tumor progression compared to the PI3K/Akt pathway, RAS/RAF/MEK/ERK acts as an important mediator of drug resistance in HER2+ BC and therapeutic targeting of this pathway may allow for overcoming resistance to HER2 targeting therapies.

### HER2/RAS/RAF/MEK/ERK targeted therapies

Current FDA-approved RAS/RAF/MEK/ERK-targeted therapies for BC (Table 2) include HER2 antibodies such as trastuzumab, pertuzumab, and margetuximab, as well as small-molecule tyrosine kinase inhibitors (TKIs) like lapatinib, neratinib, and tucatinib [150]. Additionally, antibody-drug conjugates (ADCs) like trastuzumab emtansine (T-DM1) and trastuzumab deruxtecan (T-DXd) combine anti-HER2 antibodies with cytotoxic agents, providing more treatment options [151, 152].

Before the development of HER2-targeted therapies, HER2+ BC had poor clinical outcomes due to hyperproliferative RAS/MAPK and PI3K/Akt activation [121, 124, 153]. The FDA approval of trastuzumab significantly improved survival, with early-stage HER2+ BC seeing a 50% increase in disease-free survival [154]. Trastuzumab not only inhibits ERBB receptor dimerization but also enhances cancer cell clearance through antibody-dependent cytotoxicity [155]. Although trastuzumab is currently considered one of the most effective treatments in oncology, a significant number of patients with HER2-overexpressing BC do not benefit from it, leading to the development of combination therapies. The addition of other monoclonal antibodies (mAbs), such as pertuzumab, to standard anti-HER2 therapy has led to an over 90% three-year invasive disease-free survival rate in HER2+ BC [156]. Trastuzumab and pertuzumab are widely used anti-HER2 therapies that specifically target the extracellular domain of the HER2 receptor, effectively disrupting HER2-driven signaling from the cell surface. In contrast TKIs represent another commonly used class of anti-HER2 agents, which are designed to target the intracellular kinase domain of HER2, inhibiting downstream signaling pathways that contribute to tumor growth and survival.

The development of TKIs has improved outcomes for patients with BC whose tumors develop resistance to anti-HER2 mAbs [157, 158]. These TKIs work by inhibiting the autophosphorylation of tyrosine kinases, even in the presence of ligand binding and receptor dimerization, thereby preventing further activation of the EGFR pathway [159]. In addition to the FDA-approved TKIs lapatinib, neratinib, and tucatinib (Table 2), several other TKIs are currently being investigated in clinical trials. These novel agents aim to further improve outcomes in treatment of BC, particularly in cases of resistance to existing therapies. One novel targeted therapy for HER2+ BC is an irreversible dual pan-HER TKI pyrotinib, whose efficacy and safety are evaluated in early-stage BC and metastatic setting (Table 3). Pyrotinib-containing regimens demonstrated considerable tumor response, disease control, and survival with manageable adverse effects [160].

Another pan-HER kinase inhibitor, neratinib, is under investigation in clinical trials (Table 3), though it has not yet received FDA approval for BC treatment. Combining dual-targeting approaches, like T-DM1 and neratinib—using mAbs to target the extracellular domain and TKIs for the intracellular segment—enhances the therapeutic impact, providing a more comprehensive strategy in managing HER2-positive BC (NCT05388149).

The Bruton’s Tyrosine Kinase (BTK) inhibitor ibrutinib has shown significant efficacy in targeting HER family receptors in BC. *In vitro* studies reveal that ibrutinib effectively blocks the activation of EGFR, HER2, HER3, and HER4 [161]. HER2-overexpressing BC cell lines show particular sensitivity to ibrutinib, achieving IC50 values lower than those for lapatinib, indicating enhanced efficacy at lower concentrations. Additionally, ibrutinib has been observed to inhibit cell growth, induce cell-cycle arrest, and initiate caspase-dependent apoptosis in these cell lines [162]. Currently, a phase I/II clinical trial (NCT03379428) is underway to explore the efficacy of ibrutinib in HER2-amplified metastatic BC, potentially broadening treatment options for this subtype (Table 3).

ADCs, like T-DM1 and T-DXd (Table 2), are effective for patients with progressive HER2+ BC following trastuzumab treatment [163]. Numerous ADCs are currently under investigation for BC, with several targeting HER2-positive tumors showing promising preclinical and early clinical outcomes (Table 3).

ARX788 is the next-generation, site-specific anti-HER2 ADC, that is currently studied in BC and other solid tumors. This ADC has shown considerable efficacy in preclinical studies, demonstrating activity in both *in vitro* and *in vivo* models of HER2-positive breast and gastric cancers, including those resistant to T-DM1 [164, 165]. Given the limited therapeutic options for T-DM1-resistant cancers, ARX788 is a promising candidate. The recent trial (NCT04829604) in China demonstrated that ARX788 significantly improves PFS compared to active control in patients with HER2-positive, locally advanced, or metastatic BC (Table 3). These results highlight ARX788’s potential to overcome drug resistance in HER2-positive cancers. Additionally, in 2021, the FDA granted ARX788 fast-track designation as a monotherapy for advanced HER2-positive BC in patients previously treated with HER2-targeted therapies, expediting its development as a promising therapeutic option.

IKS014 exemplifies the pursuit of safer and more effective therapies in the class of HER2-targeting ADCs (Table 3). This innovative ADC utilizes novel bioconjugation techniques and a tumor-selective linker to minimize off-target effects, thereby enhancing both safety and efficacy. Preclinical studies have shown that IKS014 demonstrates significant efficacy against HER2-positive tumor xenografts [166]. This approach reflects an ongoing effort to develop safer models with a broader therapeutic index while effectively targeting cancer cells. Another ADC, FS-1502 (Table 3), was well tolerated and demonstrated strong antitumor activity [167].

Alternative drug delivery systems, known as targeted protein degradation (TPD) technologies, have been developed in addition to traditional toxin delivery in ADCs [168]. By combining this conjugate approach with TPD, the field of degrader-antibody conjugate (DAC) has emerged, allowing for targeted protein degradation within cancer-associated cells. An example of this approach is DAC ORM-5029, represented in Table 3 [169].

RAS pathway activation varies among BC subtypes: it is high in basal-like TNBC and HER2-enriched subtypes, while luminal A and B tumors show low activation [170, 171]. TNBC, lacking clear druggable targets, remains a focus for RAS inhibition. Although BRAF alterations occur in 30% of TNBC cases, direct mutations like V600E are rare (2–3%) [117, 172]. Nonetheless, selective cases of BRAF V600E mutant TNBC have shown success with BRAF inhibitors dabrafenib and vemurafenib [173, 174]. However, the clinical relevance of BRAF targeting in BC remains unclear due to the scarcity of these mutations and the lack of specific clinical trials.

MEK inhibitors like trametinib have demonstrated preclinical efficacy, especially in TNBC, although results in ER/PR-positive and HER2-positive lines have been more modest [175]. In a clinical trial (NCT01964924) in patients with TNBC found that eight out of 37 patients in the trametinib arm experienced clinical benefits, highlighting the potential for further studies in larger cohorts to clarify MEK inhibition’s role in BC.

In metastatic HER2-positive cancer resistant to anti-HER2 therapies, somatic mutations often activate ERK/MEK signaling through the loss of *NF1,* the GTPase-activating protein that deactivates RAS [148]. This indicates that HER2 therapy-resistant BC may depend on the RAS/RAF/MEK/ERK pathway for survival, providing the rationale for combining anti-HER2 therapy with MEK inhibition. A variety of MEK inhibitors are currently being evaluated in clinical trials (Table 3).

Conversely, HER2 addiction can be induced through RTK antagonists [176]. Although these antagonists inhibit the RAS/RAF/MEK/ERK pathway, their effect on the PI3K pathway is greater, causing BC cells to increasingly rely on RAS/RAF/MEK/ERK signaling. This suggests that combining HER2 or MEK inhibitors with anti-PI3K therapy could offer a potent therapeutic approach.

For example, the imipridone ONC201, a dopamine receptor D2 inhibitor and allosteric agonist of the mitochondrial protease caseinolytic protease P, demonstrated potent synergy with trametinib in TNBC cell lines [177]. Similarly, bispecific antibodies (BsAb) like zanidatamab (NCT06435429), which target multiple residues of the HER2 receptor, have shown potential in the early phases of clinical trials by reducing mutation-mediated resistance [178].

Therapeutic advancement and improved screening techniques have drastically improved the prognosis for HER2+ BC particularly in the early stages of disease. However, primary and acquired resistance to treatment is not uncommon, especially in metastatic disease, so continued research into overcoming these resistances is warranted [153, 156].

### Limitations of current targeted therapies and strategies to overcome them in BC treatment

Targeted therapies aimed at the RAS/RAF/MEK/ERK and PI3K/Akt pathways have been pivotal in BC treatment, particularly for aggressive subtypes like HER2-positive and TNBC. However, these therapies face several significant limitations. Below are the key challenges and proposed strategies to overcome them [179].

Therapeutic Resistance: One major limitation is the development of resistance to inhibitors of the RAS/RAF/MEK/ERK and PI3K/Akt pathways, limiting the long-term efficacy of TT. Tumor cells often develop mutations in downstream signaling proteins or activate alternative pathways to bypass the effects of these inhibitors. For example, mutations in the KRAS gene or amplification of PIK3CA can result in resistance to MEK or PI3K inhibitors [180].

Combining TT, such as PI3K inhibitors with CDK4/6 inhibitors, has shown promise in overcoming resistance, especially in ER-positive BC [181] (see Table 3, NCT05508906, NCT05768139). Additionally, the combination of PI3K inhibitors with anti-HER2 therapy has demonstrated potential in enhancing efficacy in HER2-positive BC (see Table 3, NCT03765983).

Compensatory Pathway Activation: When one pathway is inhibited, tumors can activate compensatory survival pathways (Figure 1). For instance, blocking the PI3K/Akt pathway can lead to activation of the RAS/RAF/MEK/ERK pathway and vice versa [176, 182, 183]. Combination therapies targeting both pathways simultaneously, such as dual inhibition of PI3K/mTOR and MEK/ERK (NCT01160718, NCT01390818), have been proposed to suppress feedback activation [184, 185].

Tumor Heterogeneity: Intratumor heterogeneity, where different subpopulations of cancer cells respond differently to therapies, is a significant challenge in BC. Subclones of tumor cells can harbor mutations that confer resistance to PI3K/Akt or MEK inhibitors [186]. Liquid biopsies, which allow real-time monitoring of tumor evolution, are being explored to track resistance mutations (NCT05625087, NCT03881384, NCT05601440). Additionally, adaptive therapy approaches are being used to modulate treatment dosing based on tumor heterogeneity [187].

Toxicity and Side Effects: TT can cause to significant side effects. For instance, PI3K inhibitors like alpelisib are associated with hyperglycemia, while MEK inhibitors can cause ocular toxicities [96, 188].

Preclinical studies in mouse models indicate that PI3Kα inhibition reduces glucose uptake in insulin-responsive tissues such as adipose tissue and muscle, resulting in hyperglycemia and compensatory insulin release from the pancreas, which diminishes the effect of PI3K inhibition [189].

Selecting patients based on biomarkers, such as PIK3CA mutations, can help minimize unnecessary toxicity (ComboMATCH Screening Trial, NCT05564377). Additionally, exploring intermittent dosing schedules may manage these toxicities without reducing efficacy [181]. Optimizing dosing regimens, developing more selective inhibitors, and enhancing drug delivery systems are also essential for reducing adverse effects.

Limited Efficacy in Metastatic BC: In metastatic disease therapies targeting the PI3K/Akt and RAS/RAF/MEK/ERK pathways often show limited efficacy. This is especially true in TNBC, where resistance mechanisms frequently emerge [184]. Combining TT with immunotherapies, such as immune checkpoint inhibitors, is a promising strategy. Research suggests that PI3K inhibitors can modulate the tumor immune microenvironment, enhancing the effectiveness of immunotherapy [190, 191].

Lack of Predictive Biomarkers: The absence of reliable biomarkers poses a challenge in predicting which patients will respond to therapies targeting the RAS/RAF/MEK/ERK and PI3K/Akt pathways [192]. Ongoing research in genomic profiling and personalized medicine aims to identify biomarkers - such as PIK3CA mutations, PTEN loss, and KRAS/NRAS mutations - to guide therapy selection (NCT05652569, CATCH-GUIDE trial; NCT05564377, ComboMATCH Screening Trial; NCT06625775) [186, 193].

Tumor Microenvironment (TME) Resistance: TME, including stromal and immune cells, can contribute to resistance by providing growth factors that bypass inhibited pathways [194–198]. Strategies targeting the TME, such as inhibitors of cancer-associated fibroblasts (CAFs) and immune-modulatory therapies, are currently under investigation [199–202]. Combination therapies targeting both the PI3K pathway and the immune microenvironment have shown potential in clinical trials [203, 204].

Conclusion: While targeted therapies for the RAS/RAF/MEK/ERK and PI3K/Akt pathways have advanced BC treatment, addressing challenges such as resistance, toxicity, and tumor heterogeneity is critical. Promising approaches include combination therapies, biomarker-driven patient selection, and targeting the TME.

## WNT/β-CATENIN PATHWAY

### Biological significance in cancer

The Wnt/β-catenin pathway, also known as the canonical Wnt pathway, plays a crucial role in BC development and metastasis [205, 206]. Wnt/β-catenin signaling is initiated by the binding of extracellular Wnt ligands to Frizzled (FZD) or LRP5/6 transmembrane receptors, which leads to the downstream phosphorylation and nuclear localization of β-catenin [207, 208]. β-catenin mediates the activation of TCF/LEF family transcription factors, causing the transcription of Wnt/β-catenin pathway target genes [209]. In the absence of Wnt ligand binding, β-catenin is hyperphosphorylated by the destruction complex, comprised of Axin, APC, GSK-3β, and CK1α, leading to its degradation (Figure 1). While controlled regulation of Wnt signaling supports the development of healthy breast tissue, aberrant activation of the Wnt/β-catenin pathway has been described in BC, particularly TNBC, and confers a worse prognosis [210–213]. There are also multiple documented “non-canonical” Wnt pathways relevant to BC, including the Wnt-planar cell polarity (PCP) and Wnt-Ca^2+^, which lead to distinct transcriptional alterations in the cell [208].

The specific mechanisms by which this signaling axis confers cancer aggressiveness are multifaceted. For one, β-catenin can increase the expression of the oncogenic transcription factor c-Myc and the cell-cycle protein Cyclin D1 [214–216]. Conversely, c-Myc has been demonstrated to increase Wnt pathway signaling, suggesting positive feedback [217]. Further, β-catenin plays a role in the epithelial-to-mesenchymal transition (EMT), which confers migratory and metastatic potential to tumor cells. Studies have demonstrated a direct connection between Wnt/β-catenin signaling and the EMT, in which Wnt activation can decrease E-cadherin levels by upregulating its transcriptional repressor, Slug [218]. One study in mice found a causal link to metastasis, with loss of p53 causing increased neutrophilic inflammation systemically, expediting BC metastasis [219]. The Wnt/β-catenin pathway is additionally theorized to maintain cancer stem cell (CSC) populations, though its specific function in this aspect of BC is still being investigated [213, 220, 221].

β-catenin also plays complex roles in apoptosis, demonstrating differential effects with extrinsic and intrinsic apoptotic signals. On one hand, β-catenin signaling correlates with upregulation of the anti-apoptotic protein Bcl-2; on the other hand, β-catenin signaling increases sensitivity to extrinsic apoptosis by TRAIL and Fas-mediated pathways [222]. Wnt signaling has also been shown to promote the expression of anti-apoptotic markers such as survivin, supporting the survival of Wnt-addicted cancer cells [223, 224].

### Emerging therapeutic targets

Numerous strategies have been proposed to interfere with Wnt signaling and its components. One approach is targeting enzymes involved in producing or modifying Wnt ligands, including the acetyltransferase PORCN [225]. Other strategies include preventing extracellular Wnt binding by targeting FZD receptors or LRP/FZD complexes, or by sequestering Wnt ligands with decoy receptors [226, 227]. A third approach to inhibiting β-catenin signaling is enhancing the activity of the destruction complex. Molecules stabilizing a number of the destruction complex’s components, including Axin and CK1α have shown the preclinical potential to attenuate Wnt/β-catenin signaling [228, 229].

### Preclinical and clinical developments

To date, the success of Wnt inhibitors has widely been limited to preclinical experiments [230]. Clinical trials using Wnt-inhibiting compounds are limited in BC and, in other tumor types, often report severe toxicities and varied efficacies. A phase I clinical trial (NCT01351103) of the PORCN inhibitor LGK974 did not report any complete or partial responses [231]. A phase Ib trial of the FZD mAb vantictumab combined with paclitaxel in metastatic BC reported a 30.8% response rate for patients with TNBC but high incidence of bone fracture, limiting the future clinical relevance of the compound [232]. A phase I trial of the fusion protein ipafricept, which sequesters Wnt ligands, found tolerable doses but reported no complete or partial responses [233].

Targeting the Wnt pathway in cancer represents an attractive therapeutic approach. Several clinical trials are currently evaluating both canonical and non-canonical Wnt-targeting therapies in solid tumors [234]. For example, the small-molecule inhibitor PRI-724, which disrupts the interaction between β-catenin and its coactivator CREB [235], has shown good tolerance in patients with solid tumors [236]. Another potential target is tankyrase, which promotes the degradation of Axin [237]. Although tankyrase-specific inhibitors have shown promise in preclinical studies, they have not yet reached clinical trials [238–241]. The success of these trials in developing safe, effective treatments and identifying responsive patients will shape the future of Wnt-targeting therapies in BC care.

## NOTCH SIGNALING

### Biological significance in BC

The Notch signaling pathway, highly conserved across species, is upregulated in BC and linked to poor outcomes, especially in TNBC [242–244]. Notch signaling is triggered when a Notch receptor binds to a ligand (DLL or Jagged) on an adjacent cell [245]. This interaction leads to cleavage of the receptor by ADAM proteases, followed by further cleavage by γ-secretase (Figure 1), releasing the Notch intracellular domain (NICD). The NICD then translocates to the nucleus to regulate transcriptional targets like the HES and HEY protein families [245–247].

Overactive Notch signaling in BC influences cell proliferation and stemness. It regulates Cyclin D1 expression and may help maintain tumor stem cells, as evidenced by its promotion of mammosphere formation *in vitro* [248–251]. While Notch enhances Myc transcription in some cancers, direct evidence for this relationship in BC is still unclear [252].

In BC, Notch pathway aberrations often result in NICD accumulation [253]. Loss of negative regulators like Numb and GIT1, especially in ER-negative subtypes, along with FBXW7, is linked to poor outcomes [254–256]. Activating mutations and amplifications of *NOTCH* genes, more common in TNBC, further elevate Notch signaling [257].

### Preclinical and clinical advances in therapeutic strategies for BC

Studies have demonstrated a complex crosstalk between Notch and estrogen signaling in ERα-positive BC [258, 259]. Various therapeutic approaches are being developed to disrupt Notch signaling in BC [260]. γ-Secretase inhibitors block signal transduction following Notch ligand-receptor binding, but the safety of long-term treatment in combination with ET has not been thoroughly investigated [261, 262]. Monoclonal antibodies-targeting specific ligands and receptors, like Notch1 or DLL4, aim for greater tumor specificity [263, 264]. Another strategy involves drugs that disrupt the NICD transcriptional complex [265]. While γ-secretase inhibitors and Notch-targeting monoclonal antibodies have shown some success in reducing cancer stem cell populations in clinical trials, their widespread use is limited by toxicity [266, 267]. Developing better-tolerated therapies will be crucial to effectively targeting Notch in the clinic.

## DNA DAMAGE RESPONSE MECHANISMS IN BREAST CANCER

The DNA damage response (DDR) system is essential for preventing genomic instability, with impaired DNA repair increasing cancer risk. DNA repair pathways, critical for maintaining genomic integrity, counteract continuous DNA damage from both endogenous factors (e.g., oxidative stress, replication errors) and exogenous sources (e.g., radiation, chemotherapy). In BC cells, activating these repair mechanisms is crucial for addressing damage and maintaining genomic stability [268–270]. Key mechanisms include nucleotide excision repair (NER), base excision repair (BER), and non-homologous end joining (NHEJ) pathway, the homologous recombination (HR) pathway, double-strand break repair (Figure 1), which collectively mitigate mutations and prevent genomic instability [271, 272].

### NER pathway

The NER pathway repairs DNA damage from UV exposure and chemotherapy. NER consists of two major pathways: Global Genome NER, which scans the entire genome, and Transcription-Coupled NER, which targets damage during transcription. Both pathways involve key proteins like xeroderma pigmentosum group G (XPG) and excision repair cross-complementation group 1 (ERCC1). Dysregulation of these proteins can result in cancer, particularly BC. A study of BC patients identified three gene polymorphism linked to BC - *ERCC1* rs11615, *XPC* rs2228000, and *ERCC2/XPD* rs50872 [273, 274].

### BER pathway

The BER pathway addresses DNA damage caused by oxidative stress, UV radiation, and alkylating agents, which are common front-line components of cancer chemotherapy [269, 275, 276]. BER repairs small base lesions by removing damaged bases, cutting the DNA backbone, and replacing the nucleotides. Enzymes such as DNA glycosylases identify and excise damaged DNA, which is then repaired through either short or long patch mechanisms [277]. During this repair, X-Ray repair cross-complementing protein 1 (XRCC1) and poly (ADP-ribose) polymerase 1 (PARP-1) play a role in recruiting additional repair factors to facilitate the restoration of DNA integrity. Single nucleotide polymorphisms (SNPs) in BER genes, such as XRCC1 and PARP-1, have been linked to higher likelihood of developing BC [278–281].

### NHEJ pathway

NHEJ pathway repairs double-strand breaks in DNA. This repair mechanism involves several key components, including Ku70/80 heterodimer, X-Ray repair cross-complementing protein 4 (XRCC4), DNA-dependent protein kinases (DNA-PKs), the XRCC4-like factor (XLF) complex, and DNA ligase IV. SNPs in XRCC4 and Ku70 have been associated with an increased risk of BC [274, 282].

### HR pathway

BRCA1 and BRCA2 genes are essential to the HR pathway, a critical mechanism for repairing DNA double-strand breaks (DSBs). The proteins encoded by these genes form complexes with other proteins and enzymes to facilitate HR repair [283, 284]. BRCA2 is crucial for recruiting RAD51 to DNA DSBs, aiding repair. Mutations in BRCA1/2 disrupt this process, raising cancer risk [285].

TNBC, particularly the basal-like subtype, is associated with a higher incidence of BRCA1 mutations and defects in DNA repair pathways [283, 286]. While BRCA2 mutations are more commonly linked to ER-positive, and HER2-negative BCs [287, 288].

The ATM-Chk2 and ATR-Chk1 pathways play a key role in responding to DNA damage. ATM is activated by DSBs, leading to Chk2 activation, while ATR responds to SSBs and activates Chk1. ATM also helps initiate ATR activity to repair DSBs through HR, the primary mechanism for fixing DSBs and restarting stalled replication forks. HR-proficient cells can withstand PARP inhibition by repairing damage through HR. Inhibiting PARP1 leads to the buildup of DNA damage normally repaired by BER. Notably, depletion of key DNA damage proteins, including ATM, Chk1, Chk2, and p53, can bypass oncogene-induced senescence, promoting cell proliferation and transformation in oncogene-expressing cells [289].

The ATM-Chk2-p53 pathway may contribute to BC development. A large study of nearly 113,000 women, including over 60,000 patients with BC, identified gene alterations in ATM, BRCA1, BRCA2, PALB2, *BARD1, RAD51C, RAD51D,* Chk2, and TP53 as significant risk factors for BC. ATM and Chk2 alterations were particularly associated with ER/PR-positive BC [290–293]. Additionally, a study of 289 male BC patients found that elevated pATR expression, either alone or in combination with pChk2 and pATM, was linked to poorer survival outcomes [294].

### Therapeutic strategies targeting DDR

Recent advances have introduced several drugs targeting DDR pathways for BC treatment (Table 2). Olaparib, the first FDA-approved inhibitor of poly(ADP-ribose)-polymerase (PARP), was initially used for BRCA-deficient ovarian cancer and later approved for HER2-negative BC with BRCA1/2 mutations. PARP inhibitors, including talazoparib and olaparib, are effective for cancers with homologous recombination repair deficiencies [292, 295–298].

New PARP1-selective inhibitors like NMS-03305293 AZD5305 and AZD9574 aim to reduce side effects and show promise in preclinical studies. Other DDR inhibitors targeting ATM ATR and Chk1 have also shown effectiveness either alone or combined with PARP inhibitors [296, 299].

Developing resistance to PARP inhibitors is a significant obstacle in cancer treatment. Various mechanisms contribute to this resistance, including increased drug efflux, pathway dysregulation, restoration of the replication fork, and reverse mutations [269]. To overcome these challenges and improve the efficacy of PARP inhibitors, combination therapies with chemotherapies, immunotherapies, and other DNA damage response inhibitors have been investigated in clinical settings [300].

## EMERGING THERAPIES AND FUTURE PERSPECTIVES FOR THE TREATMENT OF BC

The landscape of BC treatment continues to evolve with the development of emerging therapies and innovative approaches, which mainly focused on overcoming resistance, improving precision in targeting tumors, and enhancing the immune response to cancer.

Targeted therapies, immunotherapies, and novel drug delivery systems are transforming BC care, particularly for aggressive subtypes like HER2-positive and TNBC.

ADCs represent a new horizon in treating various tumors, including BC, by selectively delivering cytotoxic agents directly to cancer cells while minimizing off-target effects. This approach enhances the therapeutic index and allows for targeted destruction of cancer cells with greater precision. Extensive development of ADCs targeting HER2, HER3, and TROP2 has shown significant promise in preclinical and clinical studies (Tables 2, 3).

An innovative therapeutic approach to overcoming cancer resistance combines protein degradation with the specificity of ADCs [301, 302]. By attaching protein degraders to antibodies, degrader-antibody conjugates (DACs) direct these degraders specifically to cancer cells, enabling the selective removal of proteins that drive cancer progression. This targeted strategy holds significant promise for enhancing treatment efficacy and reducing resistance across various cancers (Table 3, NCT05511844).

BsAbs are an emerging class of drugs in BC research [303, 304]. BsAbs are designed to recognize two specific antigens: one on the surface of tumor cells (such as HER2) and another on immune cells (such as CD47) [305]. By binding to two distinct targets, BsAbs can perform dual actions and may be more effective than traditional mAbs. Notably, the BsAb CD47/HER2 has shown promise in treating HER2+ mBC [305]. This BsAb enhances the anti-tumor immune response by targeting cancer cells and simultaneously engaging the immune system. Several BsAbs are currently being evaluated in clinical trials for mBC (see Table 3, section BsAb). BsAbs zanidatamab has demonstrated promising anti-tumor activity in HER2-positive cancers, with the potential to overcome resistance mechanisms [306].

Recent research has explored the potential of experimental vaccines against BC, demonstrating their ability to generate a robust immune response targeting key tumor proteins [307]. The concept behind cancer vaccines is to harness the autologous immune system to recognize and combat cancer cells effectively.

BC vaccines deliver antigens (Table 3), such as HER2 or related peptides derived from tumor-associated proteins, through various strategies [308]. Additionally, combining cancer vaccines with established therapies may enhance their efficacy [309, 310]. The FDA has not yet approved any vaccines to treat BC.

Another emerging therapeutic target is the DDR pathways. Targeting DDR pathways is gaining traction, particularly for cancers with deficient repair mechanisms, such as those harboring BRCA mutations. PARP inhibitors such as olaparib and talazoparib are effective in BRCA1/2-mutated BC and combining them with other therapies may improve outcomes [298].

PARP-1 is a crucial protein involved in maintaining genomic stability. As a nuclear protein, it serves as the key enzyme responsible for repairing damaged DNA [311]. Saruparib, which selectively targets PARP1 and is being evaluated in ongoing trials (NCT06380751, NCT04644068), has demonstrated superior tolerability and enhanced target engagement in preclinical and clinical studies compared to currently approved PARP inhibitors [312]. Another highly selective PARP1 inhibitor, HRS-1167 (M9466), in the ongoing trial NCT06516289 (Table 3), has demonstrated promising anti-tumor activity in pretreated patients with HRR mutations [313]. ATR and ATM inhibitors (NCT05986071) are also being studied to increase tumor sensitivity to DNA-damaging agents [314].

Epigenetic therapies targeting aberrant DNA methylation and histone modifications are under exploration in the ongoing trail NCT05633914 (Table 3), aiming to reverse changes that drive tumor progression and resistance [315–317].

Lastly but not least, theranostics and molecular imaging are emerging as tools for delivering targeted therapy while simultaneously enabling real-time monitoring of treatment efficacy [318, 319].

These innovations collectively represent significant advances in BC treatment, offering the potential for more personalized, effective, and durable therapeutic strategies.

Continued research into the intricate interactions between these pathways is crucial for the development of more effective targeted combination therapies. Innovations in therapeutic strategies, coupled with a deeper understanding of breast cancer biology, will be essential for advancing personalized medicine and improving clinical outcomes.
